# Does KarXT (xanomeline-trospium) represent a novel approach to schizophrenia management? A GRADE-assessed systematic review and meta-analysis of randomized controlled clinical trials

**DOI:** 10.1186/s12888-025-06696-5

**Published:** 2025-03-31

**Authors:** Hazem E. Mohammed, Menna A. Gomaa, Youssef Magdy Khalifa, Ahmed Ayman Shawky

**Affiliations:** 1 Faculty of Medicine, Assiut university, Assiut, 71511 Egypt; 2 Mansoura Manchester Program for Medical Education, Faculty of Medicine, Mansoura University, Mansoura, Egypt; 3 Medical Research Group of Egypt (MRGE), Cairo, Egypt

**Keywords:** Schizophrenia, KarXT, Xanomeline-trospium, Xanomeline, Trospium, Systematic review, Meta-analysis

## Abstract

**Background:**

Schizophrenia is a complex psychiatric disorder characterized by positive, negative, and cognitive symptoms. KarXT, a novel combination of xanomeline and trospium, offers potential therapeutic benefits for schizophrenia treatment by targeting muscarinic receptors and avoiding dopamine receptor blockade. We conducted a systematic review and meta-analysis to evaluate the efficacy and safety of KarXT.

**Methods:**

PubMed, Scopus, Web of Science, and Cochrane databases were systematically searched for relevant randomized controlled trials (RCTs) up to October 2024. Studies involving adult patients with schizophrenia treated with KarXT were included. Furthermore, the Grading of Recommendations, Assessment, Development, and Evaluation (GRADE) framework was used to assess evidence quality, and the risk of bias was evaluated using the Cochrane Risk of Bias 2.0 tool.

**Results:**

Four studies with 690 participants were included. KarXT significantly reduced Positive and Negative Syndrome Scale (PANSS) total scores compared to placebo (mean difference (MD): -13.77, 95% confidence interval (CI) [-22.33 to -5.20], P-value = 0.002), with significant improvements in positive and negative subscale scores. It significantly increased the incidence of achieving ≥ 30% PANSS score reduction (risk ratio: 2.15, 95% CI [1.64 to 2.84], *P* < 0.00001). Moreover, KarXT demonstrated a favorable safety profile, with side effects such as nausea and constipation being mild and transient. Notably, it was not significantly associated with weight gain or extrapyramidal symptoms, which are common with traditional antipsychotics.

**Conclusions:**

KarXT’s distinct mechanism and tolerability highlight its potential to address unmet needs in schizophrenia treatment. Future studies should explore its long-term efficacy, delayed adverse effects, and comparative effectiveness against existing therapies.

**Clinical trial number:**

Not applicable.

**Supplementary Information:**

The online version contains supplementary material available at 10.1186/s12888-025-06696-5.

## Background

Schizophrenia is a psychiatric disorder marked by positive symptoms like hallucinations, delusions, and disorganized speech, along with negative symptoms such as reduced motivation and limited expressiveness. Besides, it includes cognitive deficits that affect executive functions, memory, and mental processing [1]. Schizophrenia causes a considerable global burden, where its lifetime prevalence is approximately 0.3–0.7% [2–5]. As of 2016, there were 20 million people living with schizophrenia [1].

Cognitive deficits often emerge early in the illness and significantly impact daily functioning [6]. Patients with schizophrenia have a higher lifetime incidence of suicide, with 18–55% attempting on one or more occasions and 4–13% (a modal rate of 10%) completing suicide [7].

The dopamine hypothesis of psychosis remains one of the most longstanding theories in psychopharmacology [8]. This hypothesis primarily suggests decreased dopaminergic activity in the prefrontal cortex (linked to the negative symptoms of schizophrenia) alongside increased dopaminergic activity in the mesolimbic system (associated with the development of positive symptoms) [9].

While antipsychotic medications based on the dopamine hypothesis have shown effectiveness in treating the positive symptoms of schizophrenia, their impact on negative and cognitive symptoms remains limited [10]. Additionally, 20–30% of patients experience positive symptoms that are refractory to treatment with antipsychotics[11, 12], and side effects such as extrapyramidal motor symptoms, increased risk of dystonia [13], akathisia [14], tardive dyskinesia [15], weight gain [16], and drowsiness can lead to poor tolerability and adherence. It may also cause neuroleptic malignant syndrome (NMS) [17], which is a rare but potentially life-threatening disorder characterized by hyperthermia, muscular rigidity, autonomic dysfunction, and depressed or fluctuating levels of arousal that evolve over 24 to 72 h. Therefore, there is a strong need for treatment with alternative mechanisms, enhanced efficacy, and improved safety and tolerability compared to current treatments.

Over the past decade, additional pathophysiological mechanisms have been investigated [18], such as dysfunctions in serotonin, glutamate, gamma-aminobutyric acid (GABA), acetylcholine, norepinephrine, and cannabinoid systems [19]. Acetylcholine plays an essential role as a neurotransmitter in the body and brain, with its effects on muscarinic receptors being particularly relevant in schizophrenia [20]. The significance of cholinergic transmission [21] in the central nervous system lies in acetylcholine’s ability to regulate dopaminergic, GABAergic [22], and glutamatergic signaling [23], making the modulation of acetylcholine receptors a promising target for various neurological and psychiatric disorders.

Xanomeline is an oral muscarinic cholinergic receptor agonist that does not directly affect dopamine receptors but primarily stimulates M1 and M4 muscarinic cholinergic receptors, which have been implicated in the pathophysiology of schizophrenia [24]. Preclinical models also indicate that xanomeline selectively inhibits the firing of mesolimbic dopamine cells relative to dopamine cell bodies projecting to the striatum, which may translate to a faster onset of action than traditional antipsychotic medications and would not induce extrapyramidal side effects [25]. In contrast, trospium chloride is an oral muscarinic receptor antagonist that is unable to cross the blood-brain barrier and works by antagonizing muscarinic receptors primarily in peripheral tissues, which helps to reduce cholinergic adverse effects associated with xanomeline that may limit tolerability [26].

Xanomeline-trospium chloride (KarXT) is a combination of a muscarinic agonist (xanomeline) and a muscarinic antagonist (trospium). Approval for KarXT was based on findings from the EMERGENT clinical trials [27–29], which showed significant improvements in schizophrenia positive and negative symptoms while maintaining an acceptable safety profile. The US Food and Drug Administration (FDA) has approved xanomeline-trospium chloride capsules for oral use for the treatment of schizophrenia in adults [30]. There have been several recent reviews assessing the therapeutic role of KarXT in schizophrenia. McKenna et al. 2024 conducted a summary review by the Institute for Clinical and Economic Review, focusing on policy implications and cost-effectiveness [31]. Wright et al. 2024 have also conducted a network meta-analysis among KarXT and the other second-generation antipsychotics albeit through indirect comparisons [32].

Our study aims to provide a GRADE-assessed systematic review and meta-analysis with an exclusive inclusion of randomized controlled trials (RCTs) to allow for a direct, high-certainty conclusion regarding the efficacy and safety of KarXT. In addition, we conduct a comprehensive risk of bias assessment, ensuring a rigorous appraisal of the available evidence. We hope this research can bridge the gap by synthesizing evidence from RCTs in a stringent manner and evaluating the strength of evidence, giving clinicians a clear picture of KarXT’s benefits and limitations in schizophrenia management.

## Methods

This systematic review and meta-analysis adhered to the Preferred Reporting Items for Systematic Review and Meta-analysis (PRISMA) statement criteria [33]. The protocol was registered on PROSPERO with registration number CRD42024605928.

### Literature search and keywords

We conducted a search of PubMed, Scopus, Web of Science, and Cochrane Central Register of Controlled Trials (CENTRAL) for studies published up to October 2024. The search utilized keywords such as KarXT, xanomeline-trospium, schizophrenia, and schizophrenia spectrum disorders. Additionally, we reviewed the reference lists of pertinent reviews to identify further studies. A detailed description of the search strategy can be found in Supplementary Table S1.

### Eligibility criteria

Inclusion criteria for this systematic review were as follows: studies must be randomized controlled trials (RCTs) involving adult participants aged 18 or older, diagnosed with schizophrenia, with no limitations on the study’s publication date, location, sample size, or gender of the population. The intervention must involve the combination of xanomeline and trospium. Exclusion criteria included: (1) studies not published in English; (2) animal studies; (3) protocols, conference abstracts, reviews, theses, and oral presentations; and (4) studies that evaluated interventions other than xanomeline-trospium.

### Study selection and data extraction

After conducting our search strategy in selected databases, we removed duplicates and utilized Rayyan software for screening. Two authors conducted title-abstract screening independently according to inclusion and exclusion criteria. Studies meeting these criteria underwent full text screening. We also screened the references of the selected studies for additional studies that may not have been detected during the initial search. Any disagreement was settled by discussion. Two authors blindly extracted the data from the included studies into an online spreadsheet. Data extracted were study characteristics, baseline characteristics of the population, and outcome measures. Study characteristics included first author name, study design, study location, duration of treatment, intervention given to each group, measuring tools, and main findings. Baseline characteristics of the population included sample size, age, gender, race, body mass index (BMI), baseline Positive and Negative Syndrome Scale (PANSS) total score, PANSS negative subscale, PANSS positive subscale, PANSS Marder negative score, and Clinical Global Impression Severity (CGI-S) scores. Finally, our outcome measures were represented into:

### Primary outcome

- Reduction in PANSS (Positive and Negative Syndrome Scale) total score: it refers to the mean difference between the baseline and endpoint in the PANSS total score. PANSS is a 30-item rating scale used to grade the severity of schizophrenia. It consists of 3 subscales: Positive scale: evaluates symptoms like delusion and hallucination; negative scale: looks for symptoms like emotional blunting and social withdrawal; and general psychopathology: covers symptoms like depression and anxiety. Each one is rated on a 7-point scale (1 = absent, 7 = extreme). The higher the score, the severity of the condition is [34].

### Secondary outcomes

- Reduction in PANSS positive symptoms subscore: it refers to the mean difference between the baseline and endpoint in the PANSS positive subscale score.
- Reduction in PANSS negative symptoms subscore: it refers to the mean difference between the baseline and endpoint in the PANSS negative subscale score.
- Reduction in PANSS Marder negative factor score: due to the multifaceted nature of negative symptoms, we included the PANSS Marder negative factor score to provide a more sensitive measure of therapeutic effects. It refers to the mean difference between the baseline and endpoint in Marder negative factor score [35].
- Reduction in the GCI-S (Clinical Global Impression-Severity score) scale refers to the mean difference between the baseline and endpoint in the GCI-S score. CGI-S is a one-item scale that rates illness severity on a 7-point scale: 1 = normal, not at all ill; 2 = borderline mentally ill; 3 = mildly ill; 4 = moderately ill; 5 = markedly ill; 6 = severely ill; 7 = extremely ill [36].
- The percentage of PANSS responders refers to the number of patients who achieve ≥ 30% reduction in PANSS total score from baseline.
- Drug safety is represented by adverse effects caused by the drug like constipation, dyspepsia, nausea, vomiting, diarrhea, dizziness, and headache.
- Drug-related extrapyramidal motor symptoms are measured by the Simpson-Angus Scale, which is a 10-item rating scale used for assessing neuroleptic-induced parkinsonism in schizophrenia. It consists of one item for gait (hypokinesia), six items for rigidity, and three items for glabella tap, tremor, and salivation, respectively [37]. Akathisia was assessed by the Barnes Akathisia Rating Scale [38]. Tardive dyskinesia was assessed by the Abnormal Involuntary Movement Scale score [39].

### Risk of bias assessment

Two authors independently evaluated the risk of bias in the randomized controlled trials included in our review using the revised Cochrane Risk of Bias tool, Version 2 (ROB 2.0) [40]. Any disagreement was resolved by discussion. We assessed biases across five domains: randomization process, deviations from intended intervention, missing outcomes, and finally the measurement of outcomes. Based on the assessment, studies were classified as: low risk, some concerns, or high risk.

### Statistical analysis

We conducted the analysis with Review Manager (RevMan) Software [41]. Effect estimates of continuous outcomes (like PANSS total score) were pooled as mean difference (MD) with 95% confidence interval (95% CI), and the P-value was considered significant if it was < 0.05. A random- effect model was utilized given the small number of patients and heterogeneity. The heterogenicity of the studies was evaluated with Higgins score (I^2^), I-square ≥ 50% and chi-square P value < 0.1 indicated significant heterogeneity [42]. Furthermore, we utilized Stata software to conduct leave-one-out sensitivity analysis.

### Sensitivity analysis

To make sure that the overall evidence wasn’t dependent on a single study, we conducted sensitivity analysis in multiple scenarios, excluding one study on each scenario. We conducted a sensitivity analysis to identify the source of heterogenicity as well.

### Quality of evidence

We employed the Grading of Recommendations, Assessment, Development, and Evaluation (GRADE) tool to assess the level of the evidence certainty, which includes multiple domains: study limitations, inconsistency, risk of bias, dose-response effect, publication bias, imprecision, and plausible confounding, indirect evidence. Based on the GRADE assessment, the studies were categorized into four levels of evidence certainty: very low, low, moderate, or high [43, 44].

## Results

### Literature search

**Fig. 1:**
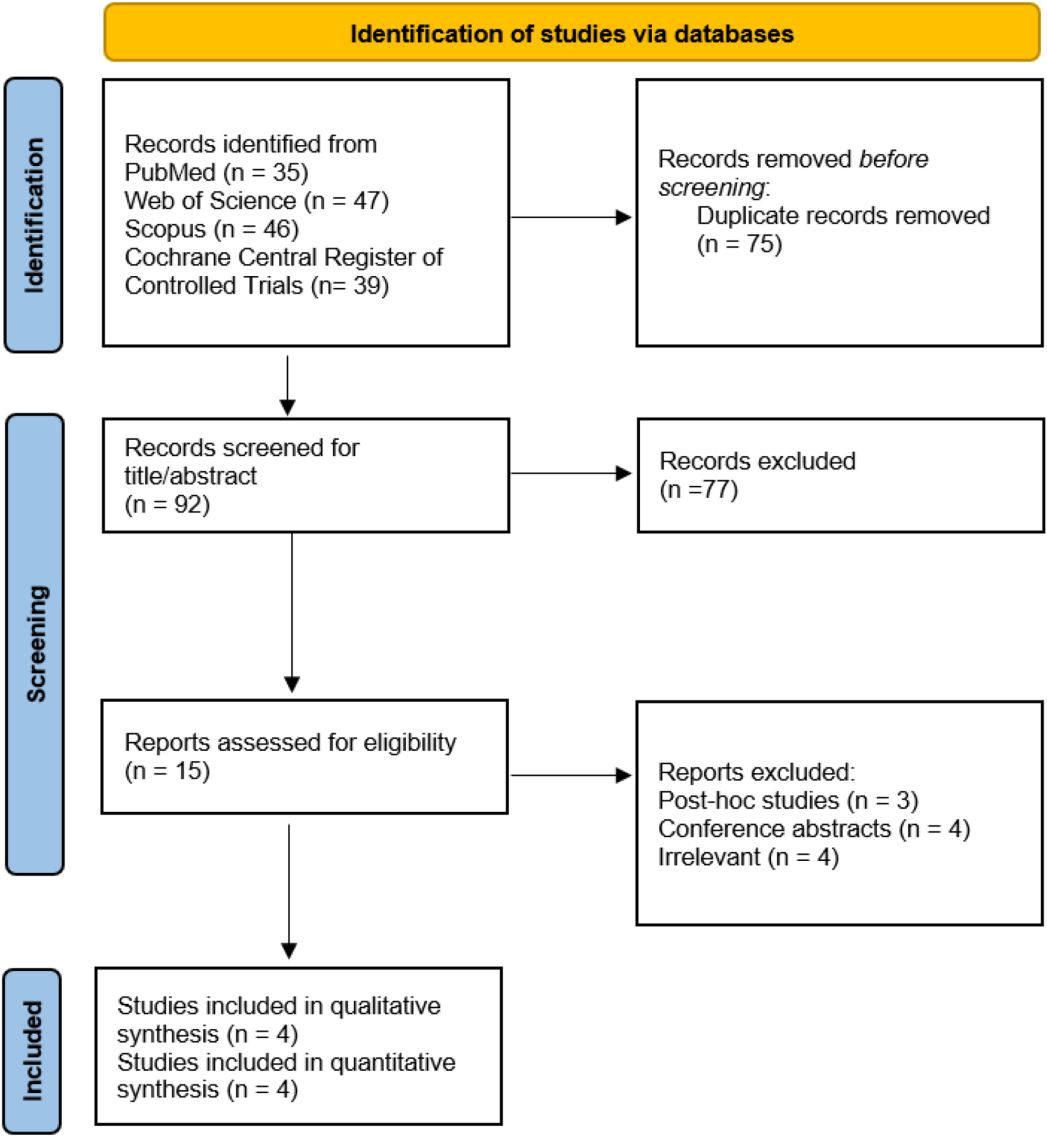
PRISMA flow diagram

We identified a total of 167 records after conducting our search strategy. After detecting 75 duplicate records, we removed them, ending with 92 records. They underwent vigorous title/abstract screening, yielding 15 records left for the full-text screening process. Eventually, a total of three RCTs and one post-hoc study were included in our qualitative and quantitative analysis. The PRISMA flow diagram is represented in Fig. 1.

### Study and population characteristics

A total of three RCTs [27–29] and one post-hoc study [45] were enrolled in our systematic review and meta-analysis. They collectively introduced a total number of 690 participants. Only one study was a post-hoc analysis study and provided further analysis regarding ≥ 30% reduction from baseline in PANSS total score, which was included in our meta-analysis [45]. Kaul et al. 2024 study (EMERGENT-3) showed the largest sample size among all studies with 256 patients [29]. The duration of treatment was comparable and equal among all RCTs. Moreover, patients were involved in studies based on diagnosis according to DSM-V. The gradual increase in dosing was the same between all studies. The mean age among patients did not vary noticeably, as it ranged between 41.6 and 46.1, with males being the most predominant patients in all studies. All study characteristics, including sample size and key findings, are represented in Table 1. Furthermore, the characteristics of the studies’ population are summarized in Table 2.

### Quality assessment

**Fig. 2:**
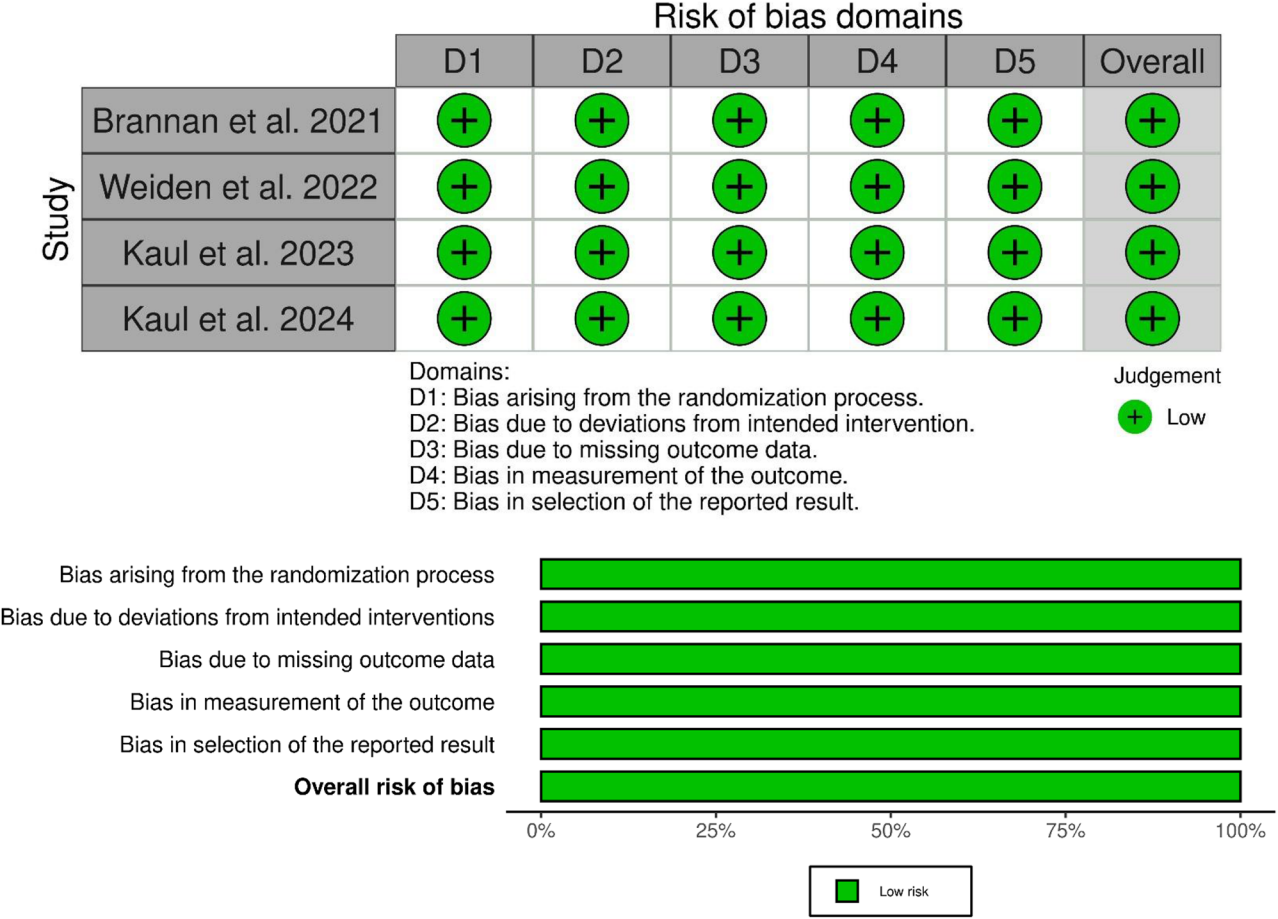
Risk of bias summary and graph

The risk of bias, evaluated using the Cochrane Risk of Bias tool version 2, is illustrated in Fig. 2. All included RCTs were determined to have a low risk across all assessed domains, resulting in an overall low risk of bias.

**Table 1:** Included studies characteristics

Study name and year	Brannan et al. 2021 (EMERGENT-1)	Weiden et al. 2022 † (post-hoc study)	Kaul et al. 2023 (EMERGENT-2)	Kaul et al. 2024 (EMERGENT-3)
Sample size	182 patients	182 patients	252 patients	256 Patients
Study design	Randomized, double-blind, placebo-controlled, phase 2 trial	Post-hoc analysis of randomized, double-blind, placebo-controlled, phase 2 study (Brannan study)	Randomized, double-blind, placebo-controlled, phase 3 trial	Randomized, double-blind, placebo-controlled phase 3 trial
Location	Twelve sites in the United States.	Twelve sites in the United States.	Twenty-two inpatient sites in the United States.	Thirty inpatient sites in the United States and Ukraine.
Duration	Five weeks	Five weeks	Five weeks	Five weeks
Population	Adults diagnosed with schizophrenia according to DSM-V	Adults diagnosed with schizophrenia according to DSM-V	Adults diagnosed with schizophrenia according to DSM-V	Adults diagnosed with schizophrenia according to DSM-V
Intervention	Days 1–2: 50 mg xanomeline and 20 mg trospium twice daily. Days 3–7: Increase to 100 mg xanomeline with 20 mg trospium twice daily. Day 8 onward: Flexible dosing, adjusting between 100–125 mg xanomeline and 20–30 mg trospium based on tolerance			
Comparator	Placebo	Placebo	Placebo	Placebo
Measuring tool	PANSS, CGI-S scale	PANSS, CGI-S scale	PANSS, CGI-S scale	PANSS, CGI-S scale
Key finding	KarXT group demonstrated a statistically significant reduction by 11.6 points (95% confidence interval [CI], − 16.1 to − 7.1; *P* < 0.001) in PANSS total score compared to placebo group.	The proportion of patients responding in the KarXT group was higher than the proportion of patients in the placebo group (*P* < 0.05 for all response criteria). The number needed to treat (NNT) (95% CI) for the number of patients needed to achieve ≥ 30% reduction in PANSS total score at week 5 was NNT = 4 (3–7).	KarXT group demonstrated a statistically significant reduction by 9.6 points (95% CI -13·9 to -5·2) in PANSS total score compared to the placebo group.	KarXT group demonstrated statistically significant reduction by 8.4 points (95% CI, − 12.4 to − 4.3; *P* < 0.001) in PANSS total score compared to placebo. Regarding CGI-S, there was a significant reduction in KarXT group compared to the placebo group (*P* < 0.001).

DSM-V: The Diagnostic and Statistical Manual of Mental Disorders, Fifth Edition; PANSS: The Positive and Negative Syndrome Scale; CGI-S: The Clinical Global Impression Scale-Severity

† Weiden et al. study is a post-hoc analysis of the EMERGENT-1 trial data and should not be interpreted as an independent randomized controlled trial

**Table 2:** Baseline characteristics of participants

Study names and groups		Brannan et al. 2021 (EMERGENT-1)		Weiden et al. 2022 † (post-hoc study)		Kaul et al. 2023 (EMERGENT-2)		Kaul et al. 2024 (EMERGENT-3)	
		KarXT	Placebo	KarXT	Placebo	KarXT	Placebo	KarXT	Placebo
Sample size of each group		90	92	90	92	117	119	125	131
Age (year), mean (SD)		43.4 ± 10.1	41.6 ± 10.1	43.4 ± 10.1	41.6 ± 10.1	45.9 (10.4)	46.1 (10.8)	43.6 (11.4)	42.6 (12.2)
Male, N (percentage)		72 (80%)	68 (74%)	72 (80%)	68 (74%)	87 (74%)	91 (77%)	87 (69.6%)	104 (79.4%)
Race, N (percentage)	Black	67 (74%)	70 (76%)	67 (74%)	70 (76%)	91 (78%)	86 (72%)	79 (63.2%)	77 (58.8%)
	White	20 (22%)	17 (18%)	20 (22%)	17 (18%)	23 (20%)	31 (26%)	45 (36.0%)	53 (40.5%)
	Asian	NA	NA	NA	NA	2 (2%)	0	1 (1%)	0
	Non-Hispanic or non-Latino ethnic	71 (79%)	79 (86%)	71 (79%)	79 (86%)	NA	NA	NA	NA
	other	3 (3%)	5 (5%)	3 (3%)	5 (5%)	1(1%)	2(2%)	NA	NA
	Not reported	NA	NA	NA	NA	NA	NA	0	1 (1%)
BMI (kg/m2), mean (SD)		28.1 (5.0)	29.6 (5.4)	28.1 (5.0)	29.6 (5.4)	30.1 (5.5)	29.1 (5.4)	28.8 (5.6)	28.0 (5.2)
PANSS total score, mean (SD)		97.7 (9.7)	96.6 (8.3)	97.7 (9.7)	96.6 (8.3)	98.2 (8.9)	97.7 (9.4)	97.3 (8.9)	96.7 (8.9)
PANSS positive symptoms subscore, mean (SD)		26.4 (3.4)	26.3 (3.2)	26.4 (3.4)	26.3 (3.2)	26.8 (3.8)	26.5 (3.7)	26.9 (3.7)	26.4 (3.3)
PANSS negative symptoms subscore, mean (SD)		22.6 (4.4)	22.8 (4.6)	22.6 (4.4)	22.8 (4.6)	22.9 (4.1)	22.9 (3.9)	22.6 (3.2)	22.0 (3.7)
Marder negative factor score, mean (SD)		22.3 (4.7)	22.3 (5.0)	22.3 (4.7)	22.3 (5.0)	22.8 (5.10)	22.5 (4.7)	22.0 (3.7)	21.8 (4.2)
CGI-S scale, mean (SD)		5.0 (0.6)	4.9 (0.6)	5.0 (0.6)	4.9 (0.6)	5.1 (0.6)	5.1 (0.6)	5.1 (0.6)	5.0 (0.6)

SD: Standard deviation; PANSS: The Positive and Negative Syndrome Scale; CGI-S: The Clinical Global Impression Scale-Severity

† Weiden et al. study is a post-hoc analysis of the EMERGENT-1 trial data and should not be interpreted as an independent randomized controlled trial

### Efficacy of KarXT

#### PANSS total score

**Fig. 3:**
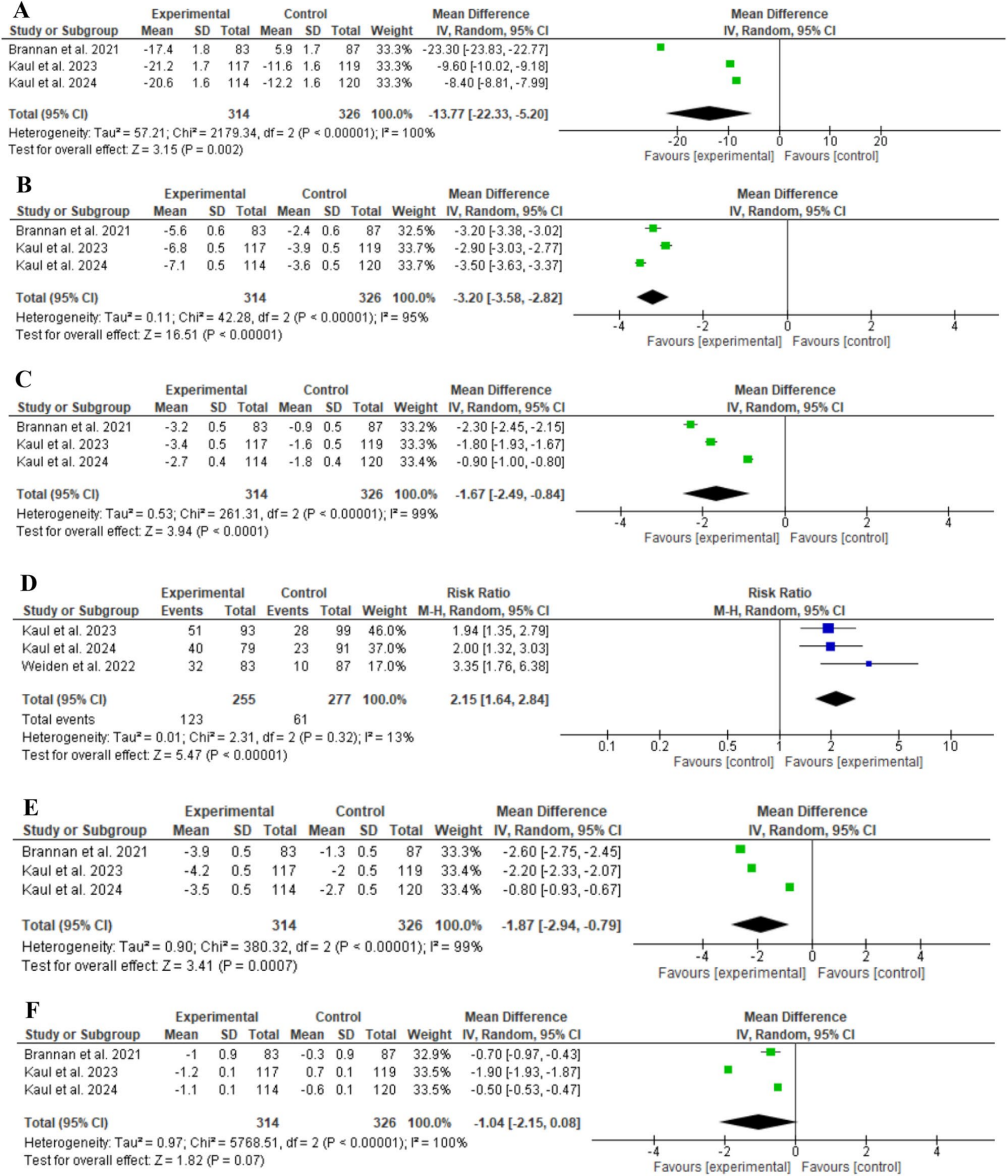
Comparison of KarXT versus Placebo in terms of **A**) PANSS total score, **B**) PANSS positive subscale, **C**) PANSS negative subscale, **D**) ≥ 30% reduction in PANSS total score, **E**) PANSS Marder negative factor score, **F**) CGI-S score

KarXT showed a statistically significant reduction in terms of PANSS total score compared to placebo (MD: -13.77, 95% CI: [-22.33 to -5.20], *P* = 0.002, as shown in Fig. 3A). However, it showed a substantial significant heterogeneity (*P* < 0.00001; I^2 = 100%).

#### PANSS subscales scores and responders

KarXT showed a statistically significant reduction with regard to both PANSS positive subscale and PANSS negative subscale scores compared to placebo (MD: -3.20, 95% CI: [-3.58 to -2.82], *P* < 0.00001, as shown in Fig. 3B), and (MD: -1.67, 95% CI: [-2.49 to -0.84], *P* < 0.0001, as shown in Fig. 3C), respectively. Both showed substantial heterogeneity (*P* < 0.00001; I^2 = 95%) and (*P* < 0.00001; I^2 = 99%), respectively. Regarding number of patients achieving ≥ 30% reduction from baseline in PANSS total score (PANSS responders), there was a statistically significant difference favoring patients receiving KarXT (RR: 2.15, 95% CI: [1.64 to 2.84], *P* < 0.00001, as shown in Fig. 3D). Furthermore, KarXT showed a statistically significant reduction in terms of PANSS Marder negative factor score (MD: -1.87, 95% CI: [-2.94 to -0.79], *P* = 0.0007, as shown in Fig. 3E), and it showed a significant substantial heterogeneity (*P* < 0.00001; I^2 = 99%).

#### CGI-S

Unlike aforementioned results, KarXT showed a statistically insignificant reduction regarding CGI-S score (MD: -1.04, 95% CI: [-2.15 to -0.08], *P* = 0.07, as shown in Fig. 3F), and it revealed significant substantial heterogeneity (*P* < 0.00001; I^2 = 100%).

### Safety of KarXT

#### Side effects

**Fig. 4:**
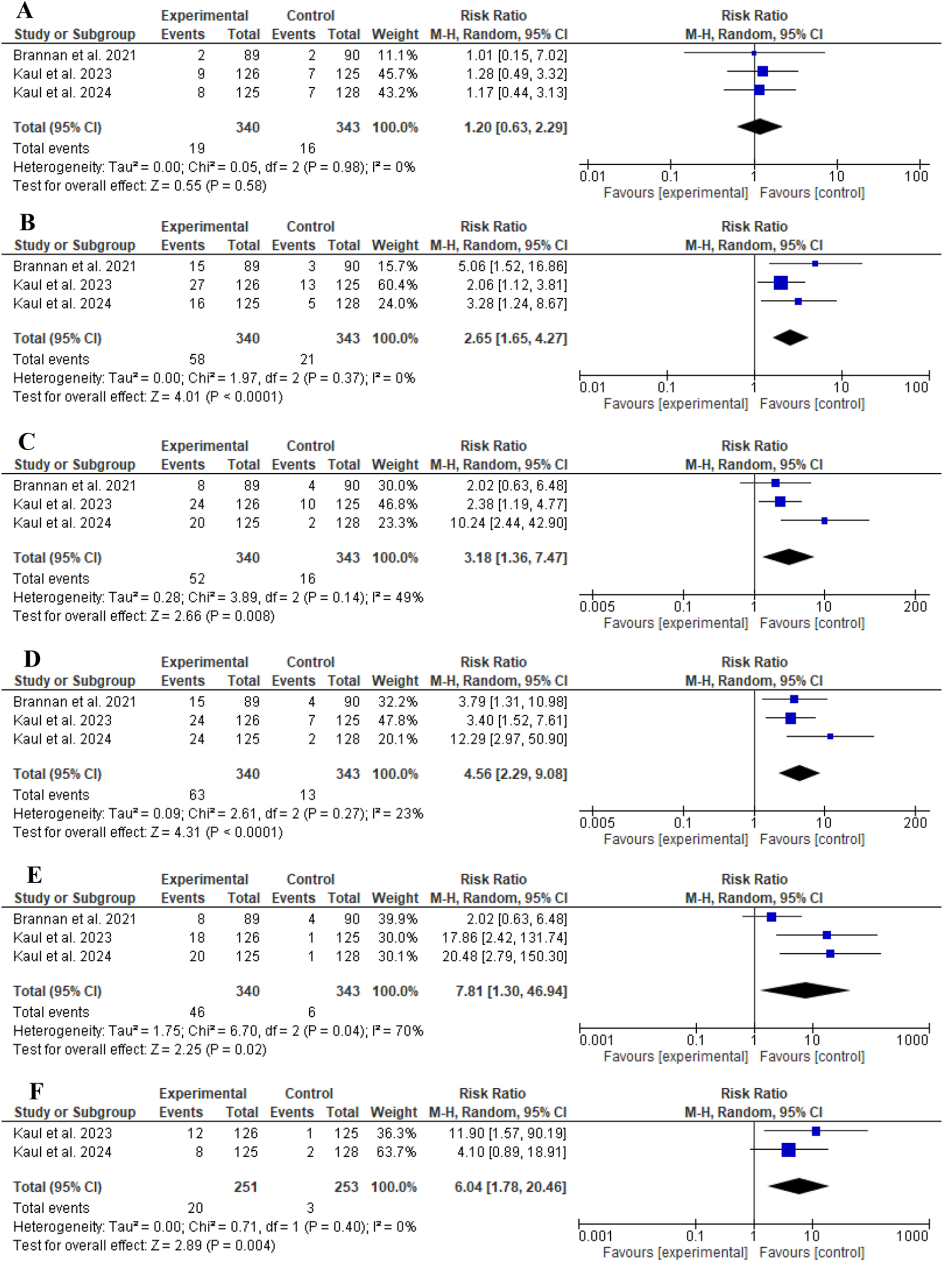
Comparison of KarXT versus Placebo in terms of **A**) Discontinuations due to adverse events, **B**) Constipation, **C**) Dyspepsia, **D**) Nausea, **E**) Vomiting, **F**) Hypertension

In general, no significant difference in discontinuations related to treatment-emergent adverse events was found between KarXT and placebo (RR: 1.20, 95% CI: [0.63 to 2.29], *P* = 0.58, as shown in Fig. 4A). Notably, nausea was the most reported side effect in the intervention group (63/340). The safety profile of KarXT showed a statistically significant incidence of constipation, dyspepsia, nausea, vomiting, hypertension, (RR: 2.65, 95% CI: [1.65 to 4.27], *P* < 0.0001, as shown in Fig. 4B), (RR: 3.18, 95% CI: [1.36 to 7.47], *P* = 0.008, as shown in Fig. 4C), (RR: 4.56, 95% CI: [2.29 to 9.08], *P* < 0.0001, as shown in Fig. 4D), (RR: 7.81, 95% CI: [1.30 to 46.94], *P* = 0.02, as shown in Fig. 4E), and (RR: 6.04, 95% CI: [1.78 to 20.46], *P* = 0.004, as shown in Fig. 4F), respectively. The remaining side effects, diarrhea, headache, and dizziness, were not significant, and their forest plots are represented in Supplementary Fig. 1.

#### Weight change and extrapyramidal side effects

**Fig. 5:**
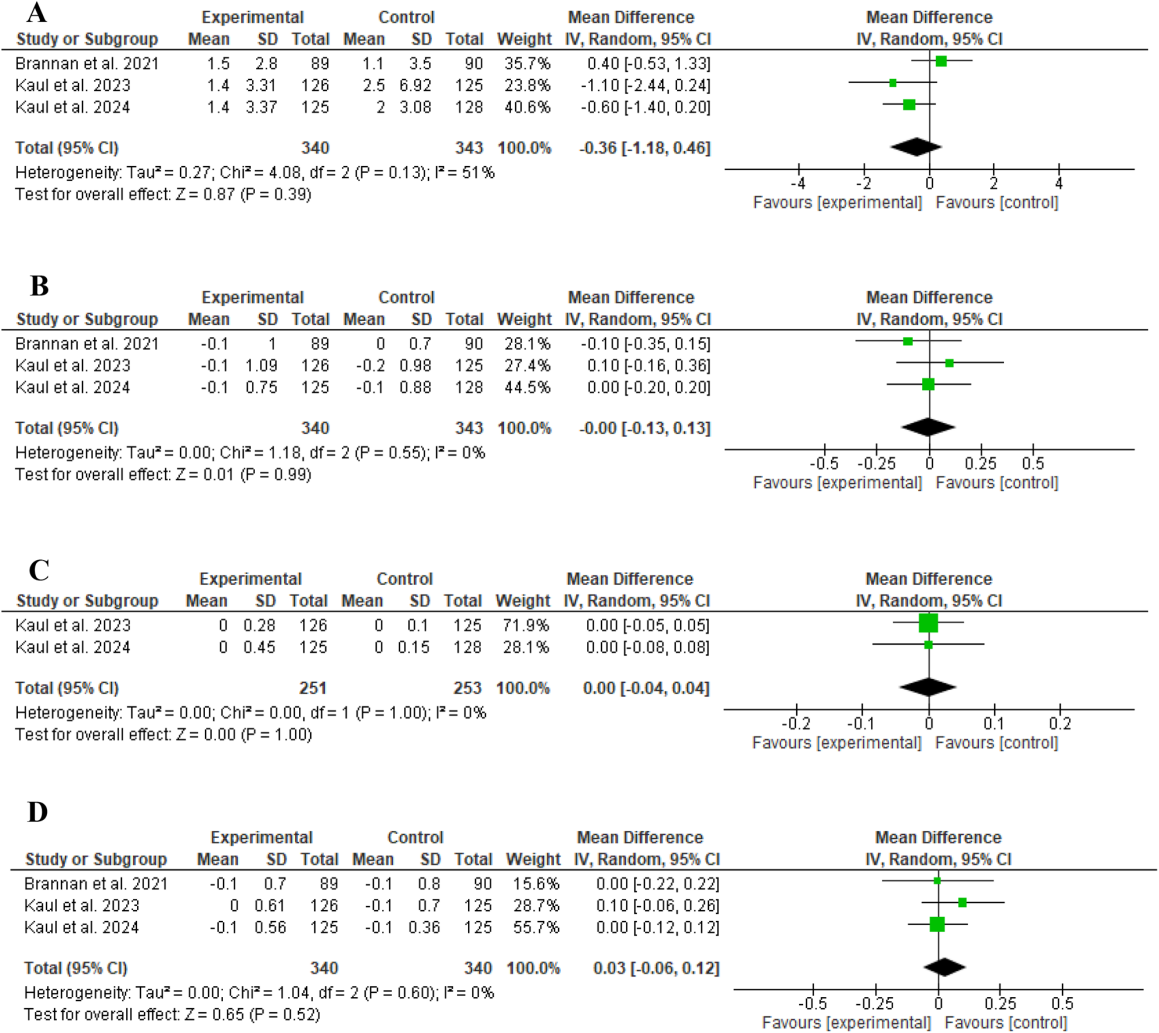
Comparison of KarXT versus Placebo in terms of **A**) Weight change, **B**) Barnes Akathisia Rating Scale, **C**) Abnormal Involuntary Movement Scale, **D**) Simpson-Angus Scale

KarXT was not significantly associated with weight gain compared to placebo (MD: -0.36, 95% CI: [-1.18 to 0.46], *P* = 0.39, as shown in Fig. 5A). Similarly, no significant difference existed between KarXT and placebo regarding the Barnes Akathisia Rating Scale, the Abnormal Involuntary Movement Scale, and the Simpson-Angus Scale, (MD: 0.00, 95% CI: [-0.13 to 0.13], *P* = 0.99, as shown in Fig. 5B), (MD: 0.00, 95% CI: [-0.04 to 0.04], *P* = 1.00, as shown in Fig. 5C), and (MD: 0.03, 95% CI: [-0.06 to 0.12], *P* = 0.52, as shown in Fig. 5D), respectively.

### Sensitivity analysis

Sensitivity analysis was conducted by excluding each study one at a time to explore sources of heterogeneity and test the robustness of the results. No single study exclusion resolved the heterogeneity in any of the heterogenous outcomes. Moreover, the pooled effect size remained stable and significant after sensitivity analysis, demonstrating that no single study disproportionately influenced the overall findings.

Interestingly, excluding either Kaul et al. 2023 (EMERGENT-2) [28] or Kaul et al. 2024 (EMERGENT-3) [29] made the CGI-S result statistically significant. All the leave-one-out sensitivity analysis plots are represented in Supplementary Fig. 2.

### Quality of the evidence

The certainty of evidence regarding KarXT efficacy in the most important and relevant outcomes was assessed using GRADE. The outcomes, including PANSS total score, PANSS positive symptoms subscale, PANSS negative symptoms subscale, and PANSS Marder negative symptoms Subscale, were downgraded at the inconsistency domain resulting in a moderate overall certainty of evidence. CGI-S was downgraded at two domains: inconsistency and imprecision, yielding a low overall certainty of evidence. A summary of the findings and a GRADE evaluation of the outcomes are shown in Table 3.

**Table 3:** Summary of findings and quality of evidence

Certainty assessment							Summary of findings		
Participants (studies) Follow-up	Risk of bias	Inconsistency	Indirectness	Imprecision	Publication bias	Overall certainty of evidence	Study event rates (%)		Anticipated absolute effects
							With Placebo	With KarXT	Risk difference with KarXT
**PANSS Total Score**									
470 (3 RCTs)	not serious	serious ^**a**^	not serious	not serious	none	⨁⨁⨁◯Moderate	239	231	MD **9 lower** (10.17 lower to 7.82 lower)
**PANSS Positive Symptoms Subscale**									
640 (3 RCTs)	not serious	serious ^**a**^	not serious	not serious	none	⨁⨁⨁◯ Moderate	326	314	MD **3.2 points lower** (3.58 lower to 2.82 lower)
**PANSS Negative Symptoms Subscale**									
640 (3 RCTs)	not serious	serious ^**a**^	not serious	not serious	none	⨁⨁⨁◯ Moderate	326	314	MD **1.67 points lower** (2.49 lower to 0.84 lower)
**PANSS Marder Negative Symptoms Subscale**									
640 (3 RCTs)	not serious	serious ^**a**^	not serious	not serious	none	⨁⨁⨁◯ Moderate	326	314	**MD 1.87 points lower** (2.94 lower to 0.79 lower)
**CGI-S**									
640 (3 RCTs)	not serious	serious ^**a**^	not serious	serious ^**b**^	none	⨁⨁◯◯ Low	326	314	MD **1.04 points lower** (2.15 lower to 0.08 higher)

RCT: randomized controlled trial; MD: mean difference; PANSS: The Positive and Negative Syndrome Scale; CGI-I: Clinical Global Impression Scale–Improvement; None: Not applicable because of the small number of included studies (egger et al.)

a. Wide variance of point estimates across studies

b. Wide 95% confidence intervals which include clinically important differences

## Discussion

This meta-analysis evaluated the efficacy and safety of KarXT (xanomeline-trospium) in treating schizophrenia, highlighting its impact on various symptomatic and safety measures. Our meta-analysis involved three main RCTs [27–29] (and one post-hoc study [45]), all RCTs published up to this study’s date. A total of 690 patients were enrolled in our meta-analysis. KarXT demonstrated a significant reduction in PANSS total score compared to placebo, with a mean difference of -13.77 points (95% CI: -22.33 to -5.20, *P* = 0.002). These findings extend to PANSS positive and negative subscales, showing respective reductions of -3.20 and − 1.67 points, both statistically significant with substantial effect sizes. Similarly, KarXT exhibited favorable results in the PANSS Marder negative factor score and the proportion of PANSS responders achieving ≥ 30% improvement. Although the RCTs included in our meta-analysis individually showed a statistically significant difference between KarXT and placebo regarding CGI-S, our pooled analysis failed to show a statistically significant difference between KarXT and placebo (*P* = 0.07).

The most frequently reported treatment-emergent adverse events (TEAEs) associated with KarXT included constipation, dyspepsia, nausea, vomiting, hypertension, and dizziness, consistent with the pharmacological activity of xanomeline and trospium on muscarinic receptors [46]. Most TEAEs emerged during the initial two-to-three weeks of treatment, were short-lived, and resolved by the conclusion of the trial [27–29]. Moreover, the intervention was not associated with a significant incidence of weight gain, and it stands out for its lack of association with extrapyramidal symptoms, a common side effect of traditional antipsychotics [47]. These results align with findings from involved trials, emphasizing KarXT’s distinctive profile compared to existing antipsychotics. Unlike first-generation neuroleptics, which have been heavily associated with extrapyramidal symptoms (EPS), affecting 61.6% of institutionalized schizophrenia patients in one study[47, 48], and some atypical antipsychotics [49], KarXT demonstrated, in our meta-analysis, no significant differences from placebo on key measures of movement disorders, including the Barnes Akathisia Rating Scale, Abnormal Involuntary Movement Scale, and Simpson-Angus Scale. This favorable safety profile, combined with its efficacy, makes KarXT a promising option for addressing unmet needs in schizophrenia management without the burden of extrapyramidal symptoms. Besides, no difference existed between the two groups in terms of treatment-emergent adverse event-related discontinuations.

Despite the encouraging efficacy data, significant heterogeneity was noted across the outcomes, with I² values consistently exceeding 95% in nearly all primary and secondary efficacy outcomes. However, sensitivity analyses confirmed the robustness of the pooled effect sizes, suggesting that the observed heterogeneity did not undermine the overall validity of the results. The substantial heterogeneity observed in PANSS outcomes warrants careful consideration. Since all the RCTs included in our meta-analysis share many main similarities, like the flexible dose design with two groups, a five-week duration of treatment, an inpatient-based setting, and comparable inclusion and exclusion criteria [27–29], it is unlikely that methodological or study design criteria factors have contributed to the observed substantial heterogeneity. Accordingly, other factors might contribute to this variability. First, differences in baseline patient characteristics, such as symptom severity and comorbidities, could influence treatment response. For instance, the included trials reported varying placebo responses, with EMERGENT-2 [28] and EMERGENT-3 [29] exhibiting a placebo response on PANSS total score (-11·6 points) and (-12.2 points), respectively, higher than that reported in the EMERGENT-1 trial (–5·9 points) [27]; and this could possibly be explained by the potential considerable expectation bias following earlier EMERGENT-1 trial results. Additionally, the multi-national nature of EMERGENT-3 (involving Ukrainian sites besides US like the previous two trials) [29] may have introduced variability in placebo responses due to differing healthcare contexts or patient support systems. Second, the slight predominance of the black population in the EMERGENT-1 (75.3%) [27] and EMERGENT-2 (75%) [28] trials, for example, compared to the EMERGENT-3 trial (61%) [29] might limit the generalizability of results to more diverse demographics and might contribute to the heterogeneity observed, where pharmacogenetic variability linked to race might contribute to differences in drug metabolism or efficacy. Lastly, differences in statistical analysis and endpoint definitions might introduce variability across studies. Future research should address these sources of heterogeneity to refine the evidence base for KarXT. Besides, the weaker negative symptom effect in EMERGENT-3 compared with EMERGENT-1 and EMERGENT-2 may be due to study population differences because EMERGENT-3 recruited U.S. and Ukrainian sites, with early termination of recruitment in Ukraine. Additionally, variability at baseline in negative symptom severity, along with the potential for delayed muscarinic modulation response, could have been a factor.

Our findings are in line with those of recent pairwise meta-analyses[50, 51], with significant improvement in overall and subscale PANSS scores with a similar safety profile. Notably, our analysis included an additional post-hoc analysis, Weiden et al. 2022 [45], of the proportion of patients with a ≥ 30% improvement in PANSS total score—a primary outcome not examined in recent meta-analyses. While a recent meta-analysis has tried to evaluate publication bias by employing funnel plots and Egger’s test [50], it is known that they have limitations and are not recommended when the number of studies included is low [52].

Interestingly, KarXT offers a novel approach to schizophrenia management by selectively targeting M1 and M4 muscarinic receptors. Unlike traditional antipsychotics, which primarily modulate dopamine pathways, KarXT avoids the dopamine blockade responsible for many adverse effects, such as extrapyramidal symptoms, weight gain, and metabolic disturbances. This unique mechanism allows KarXT to achieve significant improvements in positive and negative symptoms while minimizing these common drawbacks[53, 54]. Additionally, KarXT’s efficacy in negative symptoms, particularly the PANSS Marder negative factor, highlights its potential to address an unfulfilled need in schizophrenia treatment. Negative symptoms are notoriously resistant to traditional therapies, affecting approximately 40% of individuals [55–57]. They present a higher burden of illness compared to positive symptoms, which are often manageable with traditional antipsychotics[56, 58]. These symptoms are consistently associated with poorer functional outcomes, including impaired occupational and academic performance, reduced household integration, diminished social functioning, limited participation in activities, and a lower quality of life[56, 59]. Therefore, developing new drugs and combinations like KarXT is considered an extremely important priority in the treatment of schizophrenia. Moreover, the combination of xanomeline’s receptor specificity and trospium’s ability to mitigate peripheral side effects creates a well-tolerated therapeutic profile, with most adverse events being transient and resolving without treatment discontinuation [27–29].

This meta-analysis demonstrates several key strengths. First, it exclusively included RCTs, providing a rigorous and high-quality evidence base for evaluating KarXT’s efficacy and safety. Second, the data extraction and analysis processes were independently performed by two reviewers, with discrepancies resolved by a third reviewer, ensuring methodological transparency and reducing potential bias. Additionally, the use of sensitivity analyses enhanced the robustness of the findings, while the application of the GRADE framework offered a systematic and transparent assessment of evidence quality. Nonetheless, significant limitations must be acknowledged. The pronounced heterogeneity in efficacy outcomes introduces uncertainty regarding the consistency and generalizability of the results. Moreover, the short duration of the included trials, typically five weeks, limits the ability to draw definitive conclusions regarding the long-term efficacy and safety of KarXT. Therefore, two ongoing long-term 52-week follow-up studies [EMERGENT-4 (NCT04659174) and EMERGENT-5 (NCT04820309) trials] are anticipated to provide critical insight. Moreover, the absence of active comparator groups restricts direct comparisons between KarXT and existing antipsychotics, such as second-generation antipsychotics. According to the aforementioned limitations, we recommend that future research should prioritize longer-term RCTs with diverse populations to validate the durability and generalizability of KarXT’s effects. Trials should also include active comparators to better contextualize its efficacy and safety relative to established treatments. Additionally, subgroup analyses based on demographic and clinical characteristics could help identify patients most likely to benefit from KarXT.

## Conclusions

In our meta-analysis, KarXT demonstrated a significant reduction in efficacy outcomes, such as PANSS total and subscale scores, suggesting its potential as an effective treatment for schizophrenia. Notably, KarXT was not associated with common adverse effects typically seen with traditional antipsychotics, such as extrapyramidal symptoms, making it a promising option for alleviating schizophrenic symptoms while avoiding these detrimental side effects. Despite the observed heterogeneity across studies and the relatively short trial durations, KarXT’s novel mechanism of action and favorable safety profile highlight its potential to address critical gaps in schizophrenia management. However, further research with larger sample sizes and more extended trial periods is necessary to confirm these findings and refine their clinical application. Long-term studies are also needed to evaluate its sustained efficacy and the potential for any delayed side effects.

## Electronic supplementary material

Below is the link to the electronic supplementary material.

Supplementary Material 1

## Data Availability

Data is provided within the manuscript or supplementary information files.

## Abbreviations

BMI: Body Mass Index
CENTRAL: Cochrane Central Register of Controlled Trials
CGI: S-Clinical Global Impression-Severity
DSM: V-Diagnostic and Statistical Manual of Mental Disorders, Fifth Edition
EPS: Extrapyramidal Symptoms
GABA: Gamma-Aminobutyric Acid
GRADE: Grading of Recommendations, Assessment, Development, and Evaluation
MD: Mean Difference
NMS: Neuroleptic Malignant Syndrome
PANSS: Positive and Negative Syndrome Scale
PRISMA: Preferred Reporting Items for Systematic Review and Meta-analysis
RCT: Randomized Controlled Trial
RevMan: Review Manager (Software)
ROB 2.0: Cochrane Risk of Bias 2.0 Tool
RR: Risk Ratio
SD: Standard Deviation
TEAEs: Treatment-Emergent Adverse Events
