# Supplementary material for: Does KarXT (xanomeline-trospium) represent a novel approach to schizophrenia management? A GRADE-assessed systematic review and meta-analysis of randomized controlled clinical trials
Source: BMC Psychiatry. 2025 Mar 31;25:309. doi: 10.1186/s12888-025-06696-5 (PMC11959844; doi:10.1186/s12888-025-06696-5)
Supplement: Supplementary file 1 — Supplementary Material 1 [file 12888_2025_6696_MOESM1_ESM.docx]

| **PubMed, WOS, Scopus, and Cochrane Central Register of Controlled Trials (CENTRAL)** | (KarXT OR "xanomeline-trospium" OR LY246708 OR "Xanomeline; Trospium Chloride" OR "Karuna-Xanomeline-Trospium") AND (schizophrenia OR "schizophrenia spectrum disorders") |
| --- | --- |

**Supplementary Table (1)** Search strategy used in databases


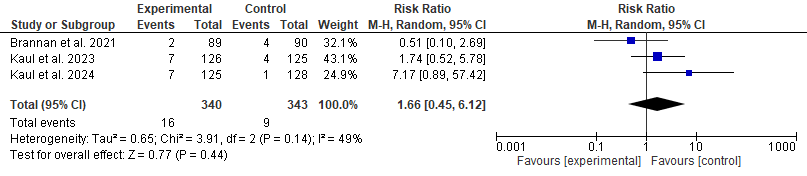

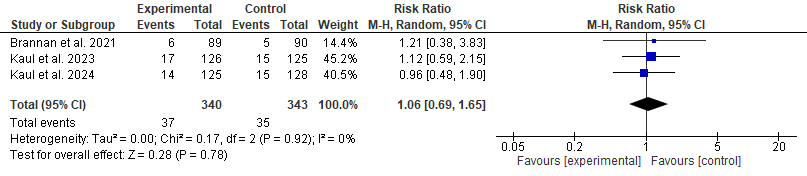

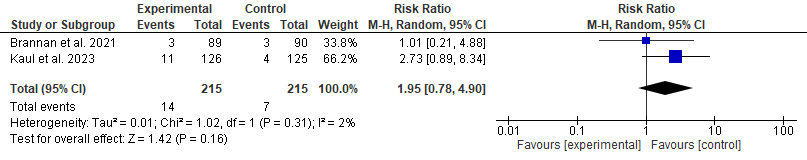


**A**

**B**

**C**

**Supplementary Figure (1):** Comparison of KarXT versus Placebo in terms of **A)** Diarrhea, **B)** Headache**, C)** Dizziness


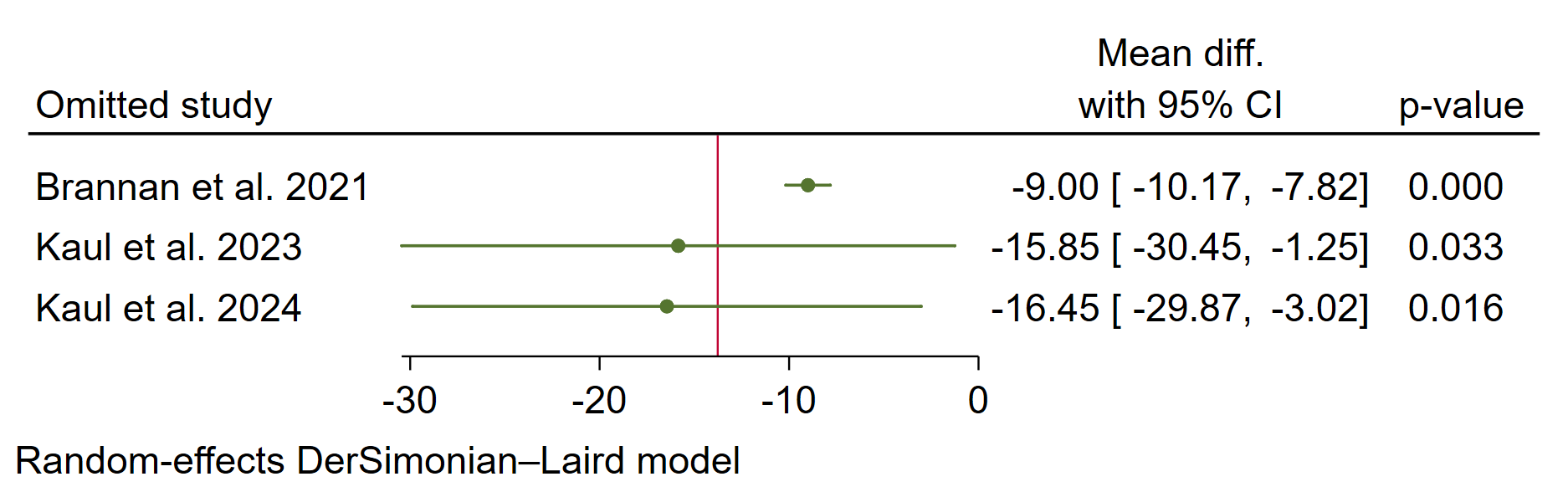

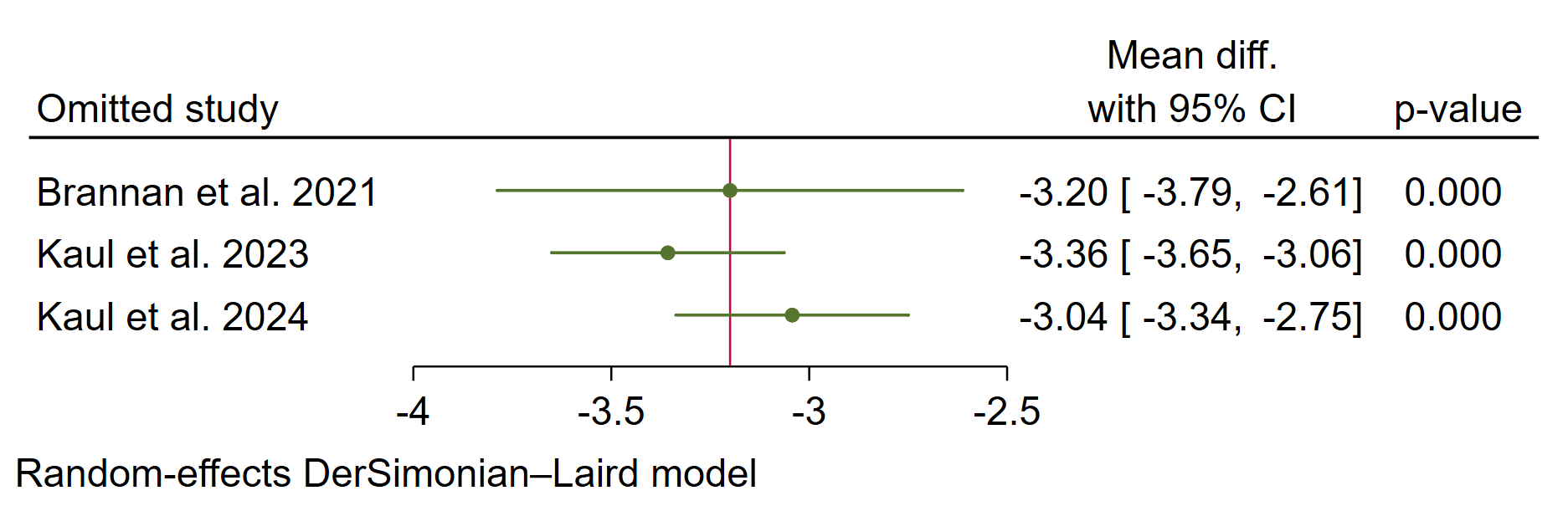

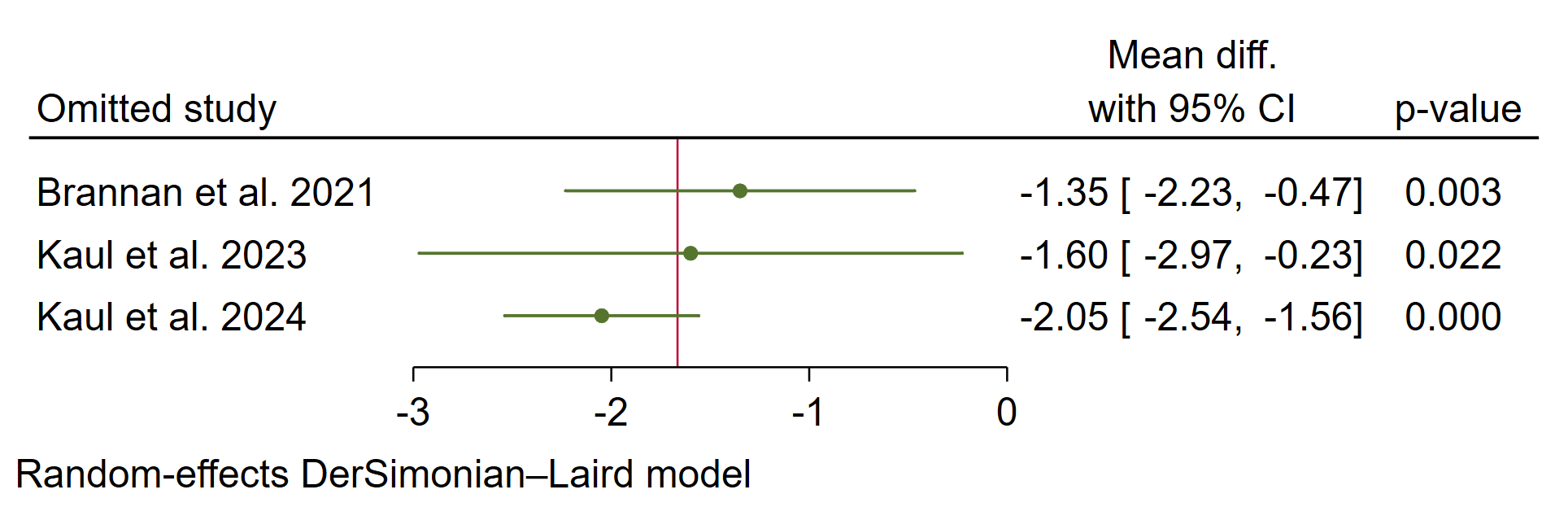

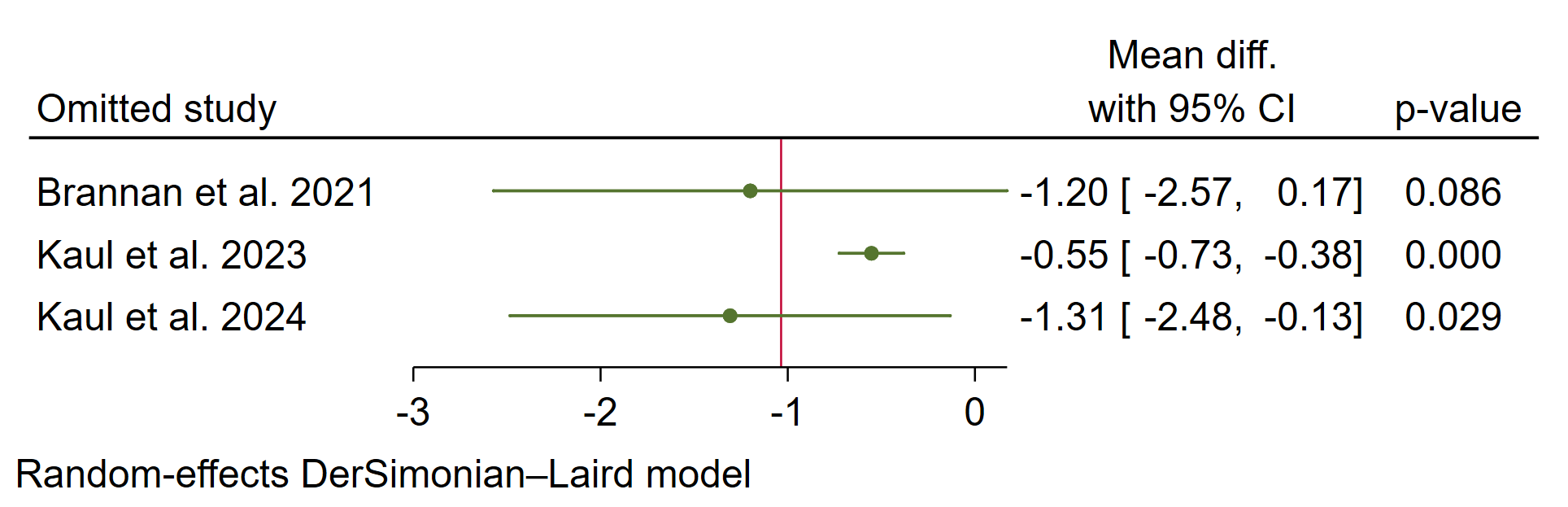


**A**

**B**

**C**

**D**

**Supplementary Figure (2):** Leave-one-out sensitivity analyses of **A)** PANSS total score, **B)** PANSS positive subscale**, C)** PANSS negative subscale, **D)** CGI-S
